# Loss of Lipocalin 10 Exacerbates Diabetes-Induced Cardiomyopathy *via* Disruption of Nr4a1-Mediated Anti-Inflammatory Response in Macrophages

**DOI:** 10.3389/fimmu.2022.930397

**Published:** 2022-06-10

**Authors:** Qianqian Li, Yutian Li, Wei Huang, Xiaohong Wang, Zhenling Liu, Jing Chen, Yanbo Fan, Tianqing Peng, Sakthivel Sadayappan, Yigang Wang, Guo-Chang Fan

**Affiliations:** ^1^ Division of Pharmaceutical Sciences, James L. Winkle College of Pharmacy, University of Cincinnati, Cincinnati, OH, United States; ^2^ Department of Pharmacology and Systems Physiology, University of Cincinnati College of Medicine, Cincinnati, OH, United States; ^3^ Department of Pathology and Laboratory Medicine, University of Cincinnati College of Medicine, Cincinnati, OH, United States; ^4^ Division of Biomedical Informatics, Cincinnati Children’s Hospital Medical Center, Cincinnati, OH, United States; ^5^ Department of Pediatrics, University of Cincinnati College of Medicine, Cincinnati, OH, United States; ^6^ Department of Cancer Biology, University of Cincinnati College of Medicine, Cincinnati, OH, United States; ^7^ The Centre for Critical Illness Research, Lawson Health Research Institute, London, ON, Canada; ^8^ Division of Cardiovascular Health and Disease, Department of Internal Medicine, University of Cincinnati College of Medicine, Cincinnati, OH, United States

**Keywords:** macrophage, lipocalin, diabetes, cardiac function, Nr4a1

## Abstract

Metabolic disorders (i.e., hyperglycemia, hyperlipidemia, and hyperinsulinemia) cause increased secretion of inflammatory cytokines/chemokines, leading to gradual loss of cardiac resident macrophage population and increased accumulation of inflammatory monocytes/macrophages in the heart. Such self-perpetuating effect may contribute to the development of cardiomyopathy during diabetes. Recent meta-analysis data reveal that lipocalin 10 (Lcn10) is significantly downregulated in cardiac tissue of patients with heart failure but is increased in the blood of septic patients. However, the functional role of Lcn10 in cardiac inflammation triggered by metabolic disorders has never been investigated. In this study, we demonstrate that the expression of Lcn10 in macrophages was significantly decreased under multiple metabolic stress conditions. Furthermore, Lcn10-null macrophages exhibited pro-inflammatory phenotype in response to inflammation stimuli. Next, using a global Lcn10-knockout (KO) mouse model to induce type-2 diabetes (T2D), we observed that loss of Lcn10 promoted more pro-inflammatory macrophage infiltration into the heart, compared to controls, leading to aggravated insulin resistance and impaired cardiac function. Similarly, adoptive transfer of Lcn10-KO bone marrow cells into X-ray irradiated mice displayed higher ratio of pro-/anti-inflammatory macrophages in the heart and worsened cardiac function than those mice received wild-type (WT) bone marrows upon T2D conditions. Mechanistically, RNA-sequencing analysis showed that Nr4a1, a nuclear receptor known to have potent anti-inflammatory effects, is involved in Lcn10-mediated macrophage activation. Indeed, we found that nuclear translocation of Nr4a1 was disrupted in Lcn10-KO macrophages upon stimulation with LPS + IFNγ. Accordingly, treatment with Cytosporone B (CsnB), an agonist of Nr4a1, attenuated the pro-inflammatory response in Lcn10-null macrophages and partially improved cardiac function in Lcn10-KO diabetic mice. Together, these findings indicate that loss of Lcn10 skews macrophage polarization to pro-inflammatory phenotype and aggravates cardiac dysfunction during type-2 diabetes through the disruption of Nr4a1-mediated anti-inflammatory signaling pathway in macrophages. Therefore, reduction of Lcn10 expression observed in diabetic macrophages may be responsible for the pathogenesis of diabetes-induced cardiac dysfunction. It suggests that Lcn10 might be a potential therapeutic factor for diabetic heart failure.

## Introduction

Diabetes mellitus (DM) is characterized by chronic low-grade inflammation in many tissues, including the heart (1, 2). It is well accepted that type 2 diabetes (T2D) is a worldwide epidemic and accounts for 90-95% of all diabetes mellitus cases (3). Diabetes mellitus predisposes affected patients to a significant range of cardiovascular complications, one of the most debilitating forms is heart failure (4). It is also referred to as diabetic cardiomyopathy (DCM) in the absence of other cardiac risk factors such as hypertension, coronary artery disease, or congenital heart diseases (5). Indeed, patients with diabetes have a 2- to 4- fold increased risk of developing heart failure than patients without diabetes and account for one-third of individuals with heart failure in clinical cases (6, 7). Despite extensive research into the pathogenesis and clinical features of diabetes-induced cardiac dysfunction in the past decades (5, 8), there are no effective treatment strategies for this condition to date.

Accumulating evidence has suggested that the impaired function of macrophages may contribute to DCM development (9, 10). Notably, macrophages are the most abundant immune cell population in human and mouse hearts and comprise two populations in general: pro-inflammatory M1-like and anti-inflammatory M2-like, which manifest distinct functions (11–13). While both M1 and M2 can clear dead cells, pro-inflammatory macrophages are crucial for clearing dead cell debris at the early stage of heart injury (14). In contrast, anti-inflammatory macrophages facilitate the resolution of inflammation and boost the repairing of injured cardiac tissue (15). As a matter of fact, recent studies have revealed that pro-inflammatory M1-like polarization is enhanced, whereas anti-inflammatory M2-like response is inhibited in diabetic hearts, resulting in cardiac inflammation and contractile dysfunction (16). Hence, manipulating the macrophage polarization profile may represent a high-yield therapeutic approach for DCM patients. Nonetheless, how to modulate the phenotypic transition of macrophages in the heart remains elusive, particularly in diabetic conditions.

Nr4a1, also known as Nur77, is a nuclear hormone receptor belonging to the NR4A subfamily (17). Unlike other well-featured ligand-activated transcription factors, Nr4a1 has long been considered as an orphan receptor due to lack of endogenous ligand for Nr4a1 identified (18). However, subsequent studies have demonstrated that unsaturated fatty acids and small synthetic molecules can bind to Nr4a1 (19, 20). In human and mouse macrophages, the expression of Nr4a1 is rapidly induced by a variety of inflammatory stimuli, such as lipopolysaccharide (LPS) and saturated fatty acids (palmitate), as well as interferon-gamma (IFN-γ) (21). Nr4a1 modulates the expression of its target genes through its transcriptional activity, which participates in a broad spectrum of biological and pathophysiological processes, including lipid homeostasis, glucose metabolism, and inflammation (19, 22). A growing body of evidence has established that Nr4a1 has potent anti-inflammatory effects (23–26). For example, Nr4a1 has been shown to limit macrophage inflammatory response through various mechanisms such as inhibition of NF-kB signaling or transcriptional reprogramming of mitochondrial metabolism (25, 26). Accordingly, murine models of Nr4a1 deficiency are more vulnerable to inflammation-driven diseases, such as sepsis and atherosclerosis (23–25). However, much less is known about the functional role of Nr4a1 in macrophages under T2D conditions.

Lipocalin 10 (Lcn10), a poorly characterized member of the lipocalin family, was initially identified as an epididymal gene since it was highly expressed in mouse epididymis (27). Subsequently, genome-wide RNA sequencing analysis of human tissues revealed that it was also expressed in other tissues such as the heart, spleen, and thyroid (28). Of note, recent studies implicate that Lcn10 may play a critical role in the pathogenesis of cardiovascular diseases (29, 30). For example, Salvo *et al.* reported that cardiac Lcn10 is significantly downregulated in patients with heart failure (29). More interestingly, using a quantitative meta-analysis of three cardiac RNA-Seq datasets, Alimadadi *et al.* reported that Lcn10 is one of the three common differentially expressed genes and is dramatically reduced by 84% in heart tissue from patients with dilated cardiomyopathy (30). Furthermore, Lahue *et al.* recently showed that Lcn10 is a candidate marker of inflammatory bowel disease, suggesting that Lcn10 may be involved in regulating inflammation (31). However, to our knowledge, the functional role of Lcn10 has never been investigated in macrophages or diabetes. Given the profound effects of macrophages in diabetes-induced cardiac dysfunction, we aimed to determine the role of Lcn10 in macrophages during T2D. Utilizing both *in vitro* and *in vivo* approaches, we found that Lcn10 deficiency promotes macrophage polarization toward a pro-inflammatory phenotype by disrupting the Nr4a1 signaling pathway, leading to exacerbated cardiac dysfunction and insulin resistance under T2D conditions.

## Materials and Methods

### Animal Models and Treatments

The Lcn10 global knockout mouse model in C57Bl/6 background was initially purchased from KOMP Repository at UC Davis (Stock # 048394-UCD). Wild-type (WT) C57BL/6 mice and CD45.1 mice (B6.SJL-Ptprca Pepcb/BoyJ) were purchased from Jackson Laboratory. Mice were housed and bred in specific-pathogen-free and temperature-controlled conditions, with 12-h light-dark cycles at the University of Cincinnati Animal Care Facility. All animal procedures followed the criteria of Care and Use of Laboratory Animals by the National Institutes of Health and approved by the University of Cincinnati Animal Care and Use Committee. To induce Type 2 diabetes, male mice (4–5 weeks old) were initially fed with a high-fat diet (HFD, Cat. # D12492, Research Diet, New Brunswick, NJ) for four weeks, followed by intraperitoneally injected with a single dose of streptozotocin (STZ, 100 µg/g body weight; Sigma-Aldrich, Cat. # S0130). Then, these mice were maintained on HFD feeding throughout the study. The onset of diabetes will be determined by a blood glucose level higher than 250 mg/dl at 48 h after STZ injection. Non-diabetic (ND) control mice were fed with a standard chow diet and received the same volume of citrate buffer injection.

### Isolation, Culture, and Treatment of Bone Marrow-Derived Macrophages

Mouse L-929 cell line was initially purchased from ATCC (CCL-1) and maintained in DMEM medium supplemented with 10% FBS, 1% penicillin/streptomycin solution, and 10 mM HEPES buffer at 37°C with 5% CO2, and 95% relative humidity. L-929 cell culture medium was collected after ten days of culture and centrifuged at 500g for 10 min, then passed through a sterile 0.45 µM filter (Millipore, Cat. # S2HVU02RE). Next, the supernatants (enriched in monocyte-colony stimulating factor, M-CSF) were aliquoted in 50 ml tubes and stored at -80 C° until use. Bone marrow-derived macrophages (BMDMs) were prepared as previously described (32, 33). Briefly, WT and Lcn10-KO mice were terminally anesthetized, and bone marrows from femur and tibia bones were flushed out using cold PBS containing 2% FBS. The marrows were filtered through a 70 µm cell strainer, followed by the removal of red blood cells (RBC) in ammonium-chloride-potassium lysis buffer (BioLegend, Cat. # 420302) for 5 min at room temperature (RT). Next, cells were grown in BMDMs complete medium (DMEM supplemented with 10% FBS, 15% L929 cell culture supernatant, 1% penicillin/streptomycin solution, 10mM HEPES buffer) and allowed to differentiate for seven days. On day 3, an additional 10 ml BMDMs complete medium was added to the culture dish. For BMDMs phenotypic polarization, cells were plated in 6-well plates with BMDMs complete medium overnight and then treated with LPS (Sigma, Cat. # L4391), IFN-γ (R&D, Cat. # 485-MI-100), Palmitate (Sigma, Cat. # P9767), oxidized Low-Density Lipoprotein (oxLDL, Invitrogen, Cat. # L34357) or IL-4 (R&D, Cat. # 404-ML-010) at the indicated doses and time points. For Nr4a1 agonist treatment, BMDMs from WT and Lcn10 KO mice were pretreated with Cytosporone B (CsnB, 5 µM) for 30 minutes, followed by LPS (10 ng/ml) + IFN-γ (10 ng/ml) stimulation at indicated time point.

### Isolation of Total RNAs for Real-Time Quantitative PCR (qRT-PCR) Analysis

qRT-PCR was conducted as previously described (34). In brief, RNAs from BMDMs or heart macrophages were extracted using miRNeasy Mini Kit (Qiagen, Cat. # 217004). According to the manufacturer’s instructions, 0.5 to 1 µg RNA was converted to complementary DNA (cDNA) using Superscript II Reverse Transcriptase (Invitrogen, Cat. # 18064014). Then the obtained cDNA products were mixed with SYBR Green Hi-ROX Master Mix (Radiant, Cat. # QS2050) and performed in triplicate using the ABI StepOnePlus Real-Time PCR System. Relative mRNA levels were normalized to GAPDH as an internal control for each sample and calculated using the delta-delta CT method (2^-ΔΔCt^). The primer sequences used for amplification are listed in **Supplementary Table S1**.

### Flow Cytometry Analysis of Macrophage Phenotype

To analyze BMDMs, cells were first incubated with CD16/32 Ab (clone 93) (eBioScience, Cat. # 14-0161-81, 1:100 dilution) to block the nonspecific binding to Fc receptors. After washing twice with FACS buffer (1XPBS without Calcium and Magnesium, 1% BSA, 1 mM EDTA), cells were stained with fluorophore-coupled antibodies for 30 minutes at 4°C. The antibodies were listed in **Supplementary Table S2**. For analysis of heart macrophages, methods were adopted and modified as previously described (35, 36). In brief, mice were anesthetized with ketamine (90 mg/kg BW) and xylazine (10 mg/kg BW), followed by perfusing with 15 ml of cold PBS *via* the left ventricle. The heart was thoroughly minced and digested in HBSS with 1 mg/mL Collagenase I (Worthington, Cat. # LS004196), 1 mg/mL Collagenase II (Worthington, Cat. # LS004177), 1 mg/mL Dispase II (Sigma, Cat. # D4693). After 45 minutes of incubation at 37°C with gentle agitation, the digested heart pieces were passed through a 40 µm cell filter to obtain a single-cell suspension, followed by centrifugation at 500g for 5 minutes at 4°C. The resulting cell pellets were resuspended in 1 ml RBC lysis buffer (BioLegend, Cat. # 420302), incubated for 3 minutes at room temperature, and washed with PBS. Following this, samples are blocked with CD16/32 Ab and stained for surface markers. The antibodies were listed in **Supplementary Table S2**. Flow cytometry was performed on a BD LSRFortessa analyzer (Research Flow Cytometry Core, Cincinnati Children’s Hospital Medical Center). The obtained data were analyzed using FCS Express V7 Software (*De Novo*, USA).

### Isolation of Macrophages From Mouse Hearts

Heart macrophages were isolated using the MagniSort™ Mouse F4/80 Positive Selection Kit (Invitrogen, Cat. # 8802-6863) according to the manual. Briefly, a single-cell suspension of the heart was prepared as described above. Then the cells were incubated with biotinylated F4/80 selection antibodies for 10 minutes at RT, followed by centrifugation at 300g for 5 minutes in a 12 X 75 mm, 5 ml tube. The cell pellets were resuspended in cell separation buffer and incubated with magnetic beads for 10 minutes at RT. The tube containing samples was placed in the magnet for 5 minutes, and the supernatant was discarded. After total three times of positive selections in the magnet and washing with cell separation buffer, cells were collected by centrifugation and ready for further experiments.

### Adoptive Transfer of Bone Marrow Cells to X-Ray Irradiated Mice

Bone marrow transplantation was performed with slight modifications (37, 38). CD45.1 male recipient mice (8-10 weeks old) were lethally irradiated with 1000 cGy (split into two doses, 500 cGy/dose, 4 hours apart) in a XenX cabinet X-ray irradiator (Preclinical Imaging Core, University of Cincinnati). Within 24 hours after irradiation, all recipient mice were retro-orbitally injected with freshly isolated bone marrow cells (~ 5 x 10^6^ cells in 150 ul of 2% FBS in PBS) from CD45.2 donor mice (WT and Lcn10-KO male mice, 4-6 weeks old). The bone marrow cells were prepared as described earlier in section 2.2 without RBC lysis. After a 2-week recovery period, during which mice were administered a chow diet (CD), mice were then fed with an HFD for 4 weeks, followed by a single dose of streptozotocin injection. Then these mice were maintained on the HFD-feeding paradigm for the indicated times until sacrifice. All recipient mice were given free access to water supplemented with 0.25 mg/ml enrofloxacin (Alfa Aesar, Cat. # J60023) one week before and four weeks after irradiation.

### Immunofluorescence Staining

For BMDMs staining, cells seeded on the coverslip were stimulated with LPS (10 ng/ml) + IFN-γ (10 ng/ml) for 1 hour. After washing with PBS two times, cells were fixed with 4% PFA for 20 minutes, followed by permeabilization in 0.3% Triton X-100 in PBS for 15 minutes at RT. Then cells were blocked with 2% BSA in PBS for 1 hour at RT. Next, samples were stained with primary Nr4a1 antibody at a dilution of 1:50 (Affinity, Cat. # DF7850) overnight at 4°C. After washing, cells were incubated with Alexa Fluor Plus 488 secondary antibody at a dilution of 1:200 (Invitrogen, Cat. # A32723) for 1 hour at RT. Finally, samples were mounted with ProLong Diamond Antifade Mounting medium with DAPI (Invitrogen, Cat. # P36962). Images were acquired on Zeiss LSM710 LIVE Duo Confocal Microscope (Live Microscopy Core, University of Cincinnati).

### Measurement of Cardiac Function by Echocardiography

Cardiac function was evaluated *in vivo* by trans-thoracic echocardiography using Vevo 2100 ultrasound imaging system (VisualSonics, FUJIFILM, Toronto, Canada) with a 40-MHz linear array transducer as previously described (39). Left ventricle (LV) end-systolic inner diameters (LVIDs), LV end-diastolic inner diameter (LVIDd), ejection fraction (EF), and fractional shortening (FS) were analyzed using Vevo 2100 analysis system with a cardiac package, and all measurements were repeated at least three times.

### Intraperitoneal Glucose Tolerance Testing (IPGTT) and Insulin Tolerance Testing (IPITT) Assays

For glucose tolerance tests, mice were fasted overnight and administered a 45% glucose solution (0.5 g/kg) intraperitoneally. For insulin tolerance tests, mice were fasted for 6 hours and then intraperitoneally injected with insulin (0.75 U/kg). Blood samples were collected from the tail vein at the indicated time points over a 2-hour period. Blood glucose levels were measured using a Contour Next EZ glucometer combined with Contour Next blood glucose test strips.

### Statistical Analysis

All data were analyzed with GraphPad Prism version 8.4 and presented as the mean ± SEM otherwise specified. A two-tailed Student’s t-test was performed when two groups were compared. Differences between more than two groups were determined by one-way or two-way ANOVA. A *p* value < 0.05 was considered statistically significant.

## Results

### The Expression of Lcn10 Is Downregulated in Macrophages Under Metabolic Stress Conditions

**Figure 1:**
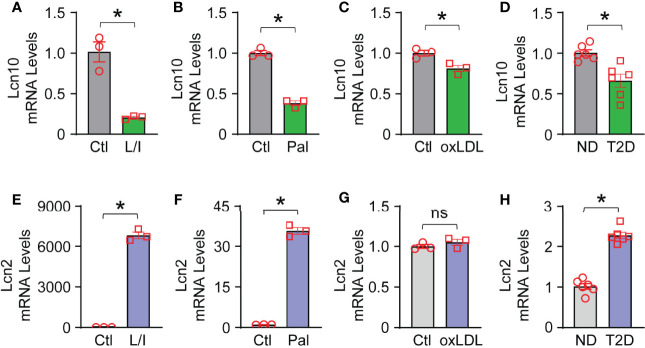
Gene expression of Lcn10 and Lcn2 in macrophages. BMDMs were treated with LPS (10 ng/ml) + IFN-γ (10 ng/ml), palmitate (0.5mM) and oxidized Low-Density Lipoprotein (10 µg/ml) for 12 hours, respectively. mRNA levels of Lcn10 **(A–C)** and Lcn2 **(E–G)** were measured by qRT-PCR (*P < 0.05, n = 3 samples per group). **(D, H)** Cardiac macrophages were isolated from the hearts of T2D mice, and gene expression of Lcn10 **(D)** and Lcn2 **(H)** were determined through qRT-PCR analysis (*P < 0.05, n = 6 mice per group). All data are presented as mean ± SEM and analyzed by student’s t-test. Ctl, control; L/I, LPS + IFN-γ; Pal, palmitate; oxLDL, oxidized Low-Density Lipoprotein; ND, non-diabetic; T2D, type 2 diabetes; ns, non significant.

To testify whether Lcn10 contributes to macrophage function, we first characterized the gene expression of *Lcn10* in macrophages in response to multiple stimuli. To mimic T2D conditions *in vivo*, BMDMs were treated with LPS + IFNγ (pro-inflammatory inducer), palmitate (a saturated fatty acid), and oxLDL (oxidized lipids) for 12 hours. We observed that the expression of *Lcn10* was downregulated by 80%, 60%, and 20%, respectively (**Figures 1A–C**), compared to controls. We also detected the expression levels of Lcn2, another lipocalin family member that is well-characterized for its role in regulating macrophage function (40, 41). Consistent with previous reports (42), the mRNA levels of *Lcn2* expression in macrophages were dramatically increased by 6800-fold and 35-fold when challenged with LPS + IFNγ or palmitate, respectively (**Figures 1E, F**); while no significant change in *Lcn2* expression was observed in the oxLDL-treated group compared to the control group (**Figure 1G**). Similar results were observed in macrophages isolated from the hearts of T2D mice (**Figures 1D, H**) compared to those from chow-fed non-diabetic (ND) mice. Collectively, these results indicate that Lcn10 may play a critical role in regulating macrophage function upon metabolic challenge.

### Lcn10 Deficiency Aggravates Macrophage Pro-Inflammatory Response Upon Multiple Stress Conditions

**Figure 2:**
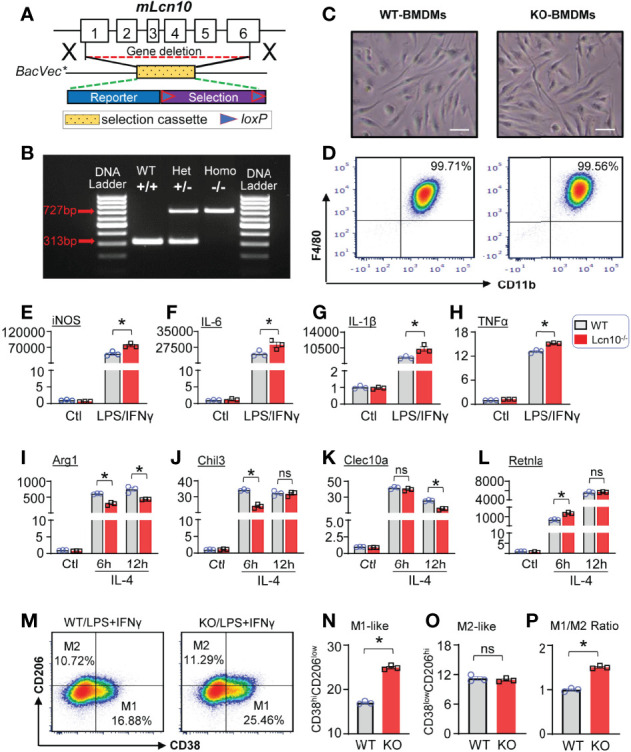
Lcn10 deficiency skews macrophages towards a pro-inflammatory phenotype. **(A, B)** Generation of the knockout mouse model of Lcn10. Genetic deletion of Lcn10 **(A)** and genotyping result **(B)**. **(C, D)** BMDMs from WT and Lcn10-KO mice were cultured and allowed to differentiate for 7 days *in vitro*. Representative images of mature BMDMs morphology **(C)** and flow cytometry plots of mature macrophage markers **(D)** from WT and Lcn10-KO mice (Scale bar, 10 µm). **(E–L)** Gene expression levels of pro-inflammatory marker genes (iNOS, IL-6, IL-1β, TNFα) **(E–H)** or anti-inflammatory marker genes (Arg1, Chil3, Clec10a, Retnla) **(I–L)** as measured by qRT-PCR in BMDMs from WT and Lcn10-KO mice stimulated with LPS (10 ng/ml) + IFN-γ (10 ng/ml) for 6 hours **(E–H)** or IL-4 (20 ng/ml) **(I–L)** for 6 and 12 hours, respectively (*P < 0.05, n = 3 samples per group). **(M–P)** Representative flow cytometry plots showing M1-like (CD38^hi^CD206^low^) and M2-like (CD38^low^CD206^hi^) **(M)** populations in BMDMs and quantification of M1-like macrophages **(N)**, M2-like macrophages **(O)**, and M1/M2 ratio **(P)** at 6 hours after LPS (10 ng/ml) +IFN-γ (10 ng/ml) treatment (*P < 0.05, n = 3 samples per group). All data are presented as mean ± SEM and analyzed by two-way ANOVA **(E–L)** or student’s t-test **(N–P)** ns, non significant.

We next went on to evaluate the functional role of Lcn10 in the regulation of macrophage polarization under various metabolic stresses. Given that lipocalin featured as a secreted protein (42, 43) and Lcn10 was downregulated as shown above, a global Lcn10-knockout (KO) mouse model was used in our study (**Figures 2A, B**). First, we isolated BMDMs from WT and Lcn10-KO mice and cultured them for 7 days *in vitro*. As shown in **Figures 2C, D**, there was no difference in morphology and differentiation of BMDMs from WT and KO mice. We then treated WT and Lcn10-KO BMDMs with LPS + IFNγ and determined the gene expression of pro-inflammatory markers using qRT-PCR. Notably, loss of Lcn10 did not alter the basal expression levels of these genes (**Figures 2E–H** and **Supplementary Figures S1A–F, S2A–I**). However, when treated with LPS + IFNγ, BMDMs from Lcn10-KO groups exhibited significantly higher expression of pro-inflammatory marker genes, including *iNOS* (**Figure 2E**), *IL-6* (**Figure 2F**), *IL-1β* (**Figure 2G**), *TNFα* (**Figure 2H**), and other pro-inflammatory genes (i.e., *CXCL1*, *CXCL9*, *CXCL10*, *IL-23*) (**Supplementary Figures S1A–F**), compared to WT groups. Similarly, KO-BMDMs showed a significantly greater inflammatory response to palmitate stimulation, as evidenced by higher mRNA levels of these pro-inflammatory genes, compared to control WT-BMDMs (**Supplementary Figures 2A–I**). In contrast, when stimulated with IL-4, an inducer of M2-like anti-inflammatory response, loss of Lcn10 impaired expression of anti-inflammatory specific genes, including *Arg1* **(** **Figure 2I**), *Chil3* (**Figure 2J**), *Clec10a* (**Figure 2K**), at 6 h or 12 h time point, whereas *Retnla* mRNA expression from KO BMDMs was increased at 6 h time point (**Figure 2L**). Furthermore, we performed flow cytometry analysis and quantified the percentage of pro-inflammatory (M1-like, CD38^hi^CD206^low^) (**Figure 2M**) and anti-inflammatory (M2-like, CD38^low^CD206^hi^) (**Figure 2M**) macrophages in response to LPS + IFNγ. In line with the gene profiling, we found that Lcn10 deletion shifted BMDMs towards a pro-inflammatory phenotype, as revealed by higher expression levels of CD38 (pro-inflammatory marker) (**Figures 2M, N**), as well as M1/M2 ratio compared with WT-BMDMs (**Figure 2P**); although no difference was observed in CD206 expression (anti-inflammatory marker) between the two groups (**Figures 2M, O**). Taken together, these results suggest that ablation of Lcn10 promotes macrophage pro-inflammatory response while inhibiting the anti-inflammatory response upon multiple stress conditions.

### Ablation of Lcn10 Leads to Exacerbated Insulin Resistance and Impaired Cardiac Function Under Diabetic Conditions

**Figure 3:**
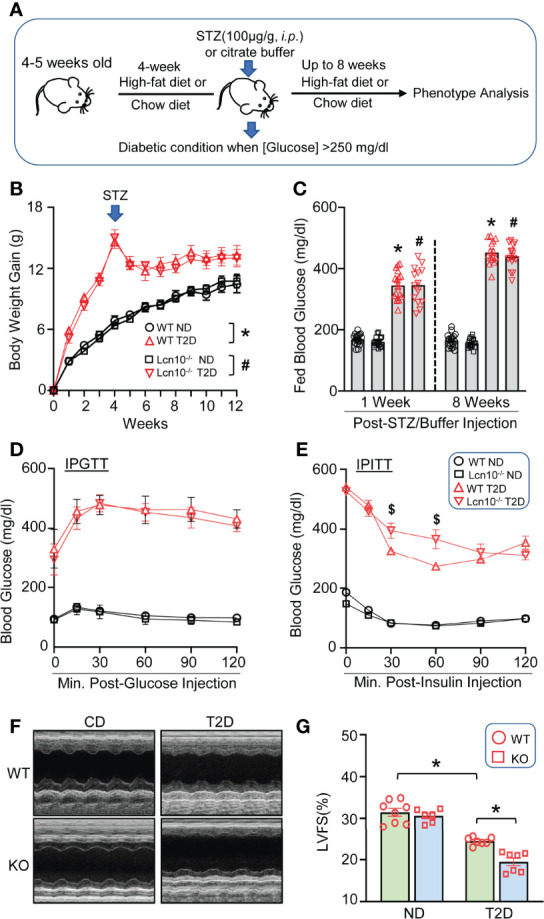
Loss of Lcn10 exacerbates insulin resistance and cardiac dysfunction under diabetic conditions. **(A)** Schematic illustration of T2D experimental design. **(B)** Body weight gain (n = 7-8 mice per group) and **(C)** fed blood glucose were determined in ND and T2D mice (n = 14-18 mice per group) (**B, C**, *P < 0.05, when comparing WT-ND to WT-T2D; ^#^ *P* < 0.05 when comparing KO-ND to KO-T2D). **(D, E)** Blood glucose level during IPGTT **(D)** (n = 6-7 mice per group) and IPITT **(E)** at 8 weeks after STZ injection (^$^ *P* < 0.05 when comparing WT-T2D to KO-T2D, n=6-8 mice per group). **(F, G)** Cardiac function was evaluated by echocardiography at 8 weeks after STZ injection (*P < 0.05, n = 6-8 mice per group). All data are presented as mean ± SEM and analyzed by two-way ANOVA. STZ, streptozotocin; LVFS, left ventricular fractional shortening; IPGTT, intraperitoneal glucose tolerance test; IPITT, intraperitoneal insulin tolerance test.

It is well established that macrophage polarization plays an essential role in metabolic disorders and concomitant cardiac dysfunction (44). We were curious to explore whether Lcn10 is involved in diabetes-induced metabolic stress. To this end, we fed WT and Lcn10-KO mice with an HFD for 12 weeks plus one dose of STZ injection at week 4 post-HFD. Mice fed with a chow diet served as controls (**Figure 3A**). Compared with chow-fed mice, HFD feeding for 4 weeks resulted in a significant increase in body weight gain in both WT and Lcn10-KO mice (**Figure 3B**). Consistent with previous reports (45, 46), STZ injection resulted in a moderate weight loss. Accordingly, blood glucose levels in the T2D group increased dramatically after STZ administration when compared to non-diabetic (ND) mice (**Figure 3C**). Interestingly, compared with WT mice, systemic Lcn10 deletion had no significant effect on body weight gain, fed blood glucose, and glucose tolerance in ND and T2D status (**Figures 3B–D**). However, KO-T2D mice clearly displayed impaired insulin sensitivity, as revealed by higher blood glucose levels during the insulin tolerance test compared with WT-T2D controls (**Figure 3E**). Importantly, this effect is independent of body weight gain, indicating that metabolic stress induced by HFD feeding and STZ injection was required for Lcn10 to influence metabolic hemostasis. Lastly, we assessed cardiac function at 8 weeks post-STZ injection using echocardiography. We found that Lcn10-KO mice exhibited normal cardiac function compared with WT controls under chow diet feeding (**Figures 3F, G**). However, compared to WT-T2D mice, KO-T2D mice showed a reduced cardiac contractile function, as evidenced by a 20% reduction in fractional shortening (**Figures 3F, G**). Collectively, these data demonstrate that Lcn10 deficiency leads to aggravated insulin resistance and cardiac dysfunction under diabetic conditions.

### Loss of Lcn10 Promotes Macrophage Pro-Inflammatory M1-Like Phenotype in T2D Hearts

**Figure 4:**
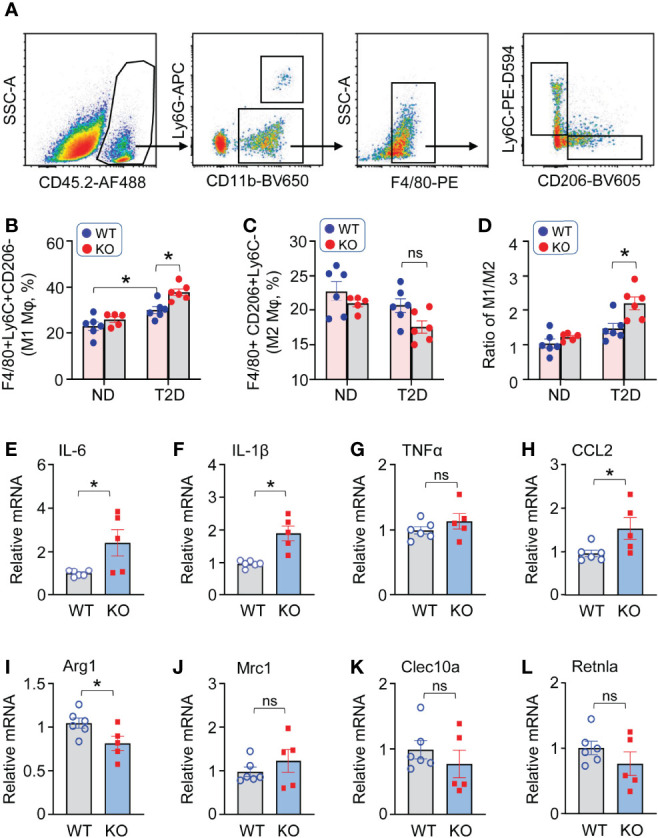
Lcn10 deficiency promotes macrophage pro-inflammatory M1-like phenotype in T2D hearts. **(A–D)** Cardiac macrophage phenotypes were determined in the heart of ND and T2D mice at 1-week post-STZ. Representative flow cytometry plots and gating strategy were shown for cardiac macrophages **(A)**, quantification of cardiac pro-inflammatory M1-like macrophages (F4/80+Ly6C+CD206-) **(B)**, anti-inflammatory M2-like macrophages (F4/80+Ly6C-CD206+) **(C)** and the ratio of M1/M2 **(D)** (*P < 0.05, n = 5-6 mice per group). **(E–L)** qRT-PCR analysis of mRNA expression of pro-inflammatory marker genes (IL-6, IL-1β, TNFα, CCL2) **(E–H)** and anti-inflammatory marker genes (Arg1, Mrc1, Clec10a, Retnla) **(I–L)** in cardiac macrophages isolated from the heart of WT and Lcn10-KO mice at 1 week post-STZ injection (*P < 0.05, n = 5-6 mice per group). All data are presented as mean ± SEM and analyzed by two-way ANOVA **(B–D)** or student’s t-test **(E–L)** ns, non significant.

Given that the imbalance of macrophage M1/M2 status may contribute to cardiac injury, we next aimed to determine the phenotypes of cardiac macrophages in T2D mice. At the early stage of diabetes (1 week after STZ administration), the flow cytometry results revealed that macrophages in KO-T2D hearts exhibited a more robust pro-inflammatory signature, as evidenced by a higher ratio of Ly6C+ population to CD206+ population compared to WT-T2Ds (**Figures 4A–D**). Such increased inflammatory phenotype in KO-T2D macrophages persisted into the late stage of diabetes, similar results of higher Ly6C+ population and lower CD206+ population were observed at 8 weeks after STZ injection (**Supplementary Figures S3B–E**), which may contribute to the development and progression of cardiac dysfunction under T2D conditions (**Figures 3F, G**). To further confirm the polarized state of cardiac macrophages, we isolated these cells from KO-T2D mice and WT-T2D control mice *via* magnetic cell sorting, and total RNA was extracted to conduct qPCR for pro- and anti-inflammatory genes. Consistent with our flow cytometry results, macrophages from KO-T2D hearts displayed increased *IL-6* (**Figure 4E**), *IL-1β* (**Figure 4F**), and *CCL-2* (**Figure 4H**) expression than those from WT-T2D controls. Interestingly, the expression of anti-inflammatory genes exerted a discrepancy pattern. The mRNA levels of *Arg1* (**Figure 4I**) in macrophages from KO-T2D hearts were decreased when compared to WT- T2D groups, while the levels of other anti-inflammatory genes, including *Mrc1*, *Clec10a*, and *Retnla*, were comparable in macrophages from WT- T2D and KO-T2D hearts (**Figures 4J–L**). Altogether, these data suggest that ablation of Lcn10 aggerates macrophage polarization to M1-like phenotype in T2D hearts.

### Adoptive Transfer of Lcn10-KO Bone Marrow Cells Impairs Cardiac Function Mainly Through Augmented Infiltration of Pro-Inflammatory Macrophages in T2D Hearts

**Figure 5:**
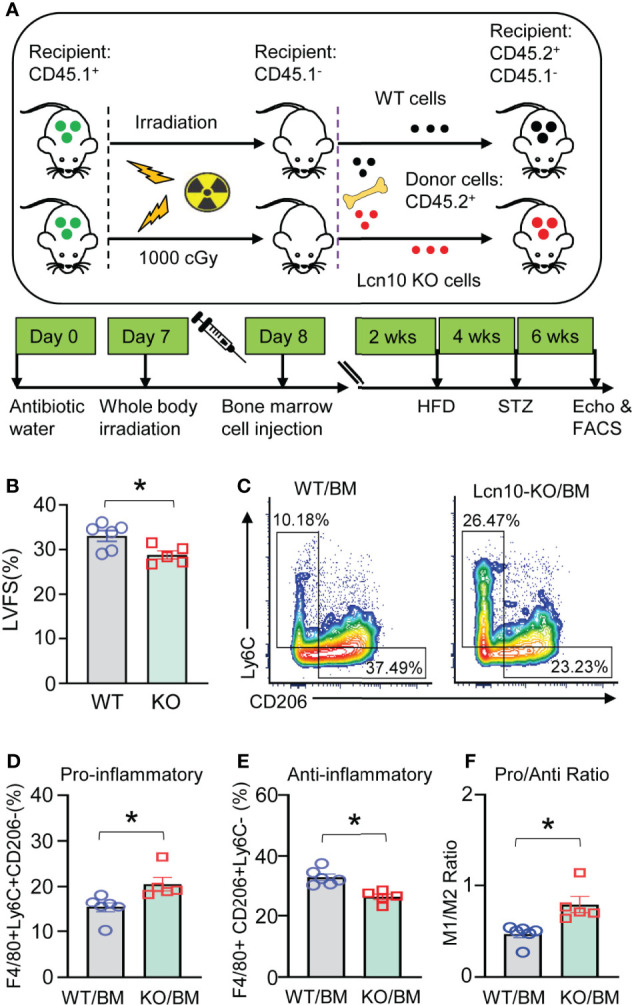
Transplantation of Lcn10-KO bone marrow cells promotes more infiltration of pro-inflammatory macrophages in T2D hearts and results in worsened cardiac dysfunction. **(A)** Graphic scheme of bone marrow cell transplantation experiments. **(B–F)** LVFS **(B)** and cardiac macrophage phenotype **(C–F)** were determined 6 weeks after STZ administration (*P < 0.05, n = 5-6 mice per group). All data are presented as mean ± SEM and analyzed by student’s t-test.

Global ablation of Lcn10 might affect the functions of other vital organs such as the pancreas, liver, or kidney, which may contribute to aggravated diabetic complications. Thus, to further clarify whether Lcn10 deficiency-elicited effects in the heart are mediated by hematopoietic cells or other cells, we performed bone marrow cell transplantation experiments. Specifically, recipient mice received whole-body irradiation to eliminate endogenous hematopoietic cell precursors and were transplanted with bone marrow cells from intact WT and Lcn10-KO mice. Then, these mice were subjected to T2D induction by the combination of HFD and STZ injection (**Figure 5A**). In line with our above results that Lcn10-KO mice displayed exacerbated diabetes-induced cardiac dysfunction, transplantation of Lcn10-deficient bone marrow cells aggravated cardiac dysfunction of recipient mice, evidenced by a 12% decrease in fractional shortening (**Figure 5B**). In addition, transplantation did not affect the percentage of neutrophils (CD45.2+CD11b+Ly6G+) in both recipient groups (**Supplementary Figures S4C, D**). However, repopulating recipient mice with Lcn10-KO cells resulted in an increased number of Ly6C+CD206- pro-inflammatory macrophages in the heart (**Figures 5C–F**). Taken together, these results suggest that Lcn10-KO hematopoietic cells are sufficient to exacerbate diabetes-induced cardiac dysfunction, mainly by triggering more infiltration of pro-inflammatory macrophages in the diabetic heart.

### Gene Enrichment Analysis of Lcn10-KO BMDMs Reveals the Nr4a1 Signaling Is Involved in Lcn10-Mediated Macrophage Polarization

**Figure 6:**
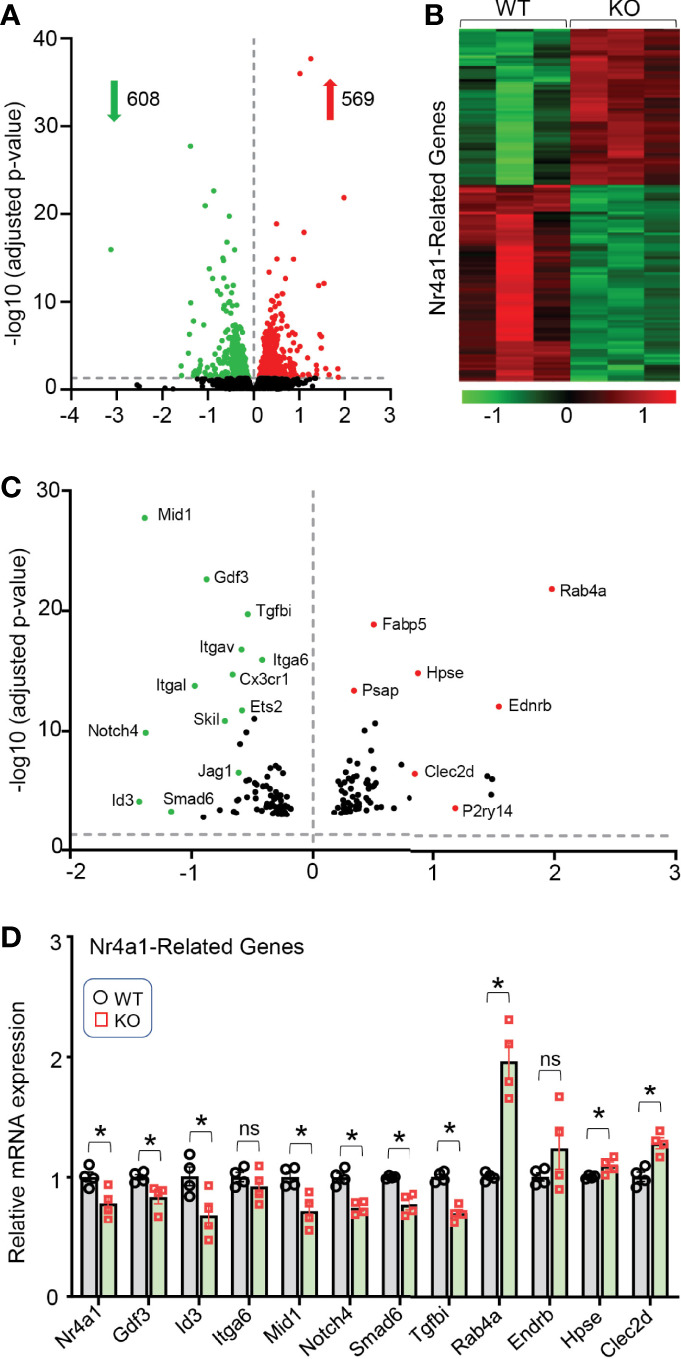
Gene expression profile in Lcn10 KO BMDMs determined by high-throughput RNA sequencing. **(A)** Volcano plot of the overall gene expression alteration in BMDMs isolated from WT and Lcn10 KO mice (n = 3 per genotype). **(B, C)** Heatmap **(B)** and Volcano plot **(C)** show many of the most differentially expressed genes are Nr4a1-related genes. **(D)** The altered expression of Nr4a1-related genes was validated by qRT-PCR (*P < 0.05, n = 4 samples per group). All data are shown as mean ± SEM and analyzed by student’s t-test ns, non significant.

To gain further insights into how the loss of Lcn10 in macrophages induces M1- like phenotype, we isolated BMDMs from WT and Lcn10-KO mice and performed RNA-sequencing analyses. Importantly, we identified that 569 genes were significantly upregulated, whereas 608 genes were remarkably downregulated in KO macrophages compared to WT counterparts (**Figure 6A**). Of interest, further analysis revealed that many of the significantly differentially expressed genes (DEGs) are directly or indirectly regulated by the Nr4a1 signaling pathway (**Figures 6B, C**). More intriguingly, we observed that the gene expression of *Nr4a1* itself was significantly decreased in KO macrophages. Notably, it is well appreciated that Nr4a1 acts as a transcriptional activator or repressor depending on post-translational modifications and coregulator protein recruitment (47, 48). In accordance with the RNA-seq data, qRT-PCR analysis further validated the altered expression of Nr4a1-related genes, such as *Gdf3*, *Mid1*, *Id3*, *Tgfbi*, and *Rab4a* (**Figure 6D**). Collectively, these data suggest that loss of Lcn10 could disrupt the Nr4a1 signal in macrophages.

### Nr4a1 Agonist Attenuates Pro-Inflammatory Response and Partially Improves Cardiac Function in Lcn10-KO Mice Under T2D Conditions

**Figure 7:**
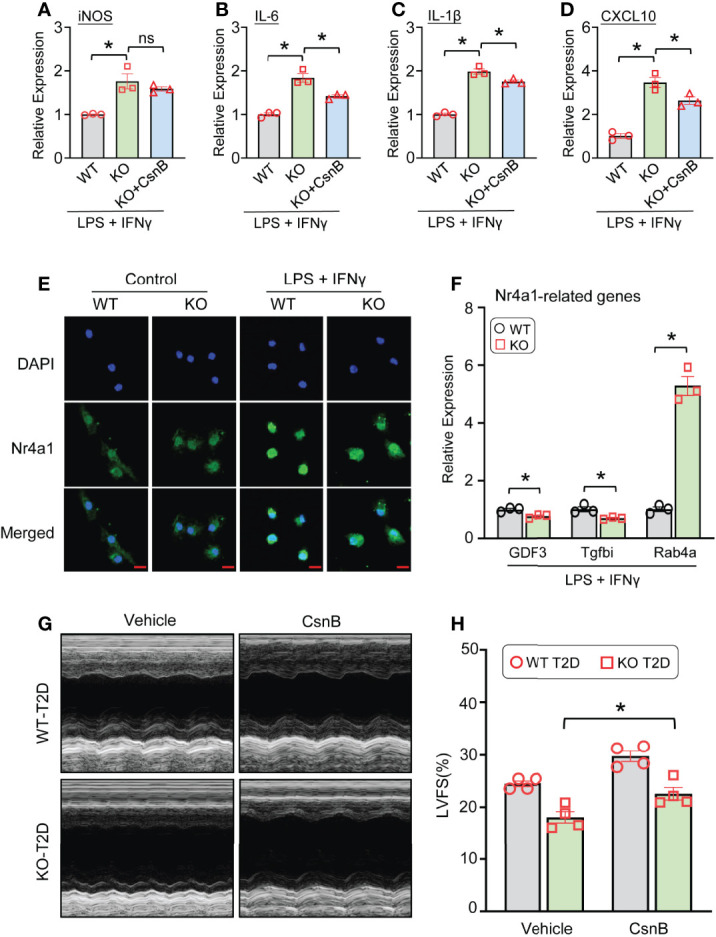
Nr4a1 agonist attenuates pro-inflammatory response and partially improves cardiac function in Lcn10-KO mice under T2D conditions. **(A–D)** BMDMs were pre-treated with CnsB (5 µM) for 30 minutes, followed by stimulation of LPS (10 ng/ml) +IFN-γ (10 ng/ml) for 6 hours. Gene expression of pro-inflammatory marker genes (iNOS, IL-6, IL-1β, CXCL10) was determined by qRT-PCR (*P < 0.05, n = 3 samples per group). **(E)** Representative images of immunofluorescence staining for Nr4a1 (green) in WT and Lcn10-KO BMDMs at 1 hour after LPS (10 ng/ml) +IFN-γ (10 ng/ml) treatment (Scale bar, 10 μm). **(F)** qRT-PCR analysis of Nr4a1-targeted genes in BMDMs after stimulation with LPS (10 ng/ml) +IFN-γ (10 ng/ml) for 3 hours (*P < 0.05, n = 3 samples per group). **(G, H)** At 1 week before STZ injection, WT and Lcn10-KO mice were injected with CsnB (5 mg/kg of BW, *i.p.*) every 3 days for total 7 weeks; then, the cardiac function was evaluated by echocardiography (*P < 0.05, n = 4 mice per group). All data are shown as mean ± SEM and analyzed by student’s t-test **(A–D, F)** or two-way ANOVA **(H)**.

Given that Nr4a1 may contribute to the Lcn10-elicited polarization in macrophages, we therefore pre-treated Lcn10-KO BMDMs with CsnB, a specific agonist of Nr4a1, 30 minutes before LPS+IFNγ stimulation. Subsequently, the gene expression of pro-inflammatory markers was analyzed. As expected, we observed that treatment of Lcn10-KO macrophages with CsnB significantly inhibited pro-inflammatory response upon LPS+IFNγ stimulation, compared to controls, as measured by mRNA levels of *iNOS*, *IL-6*, *IL-1β*, and *CXCL10* (**Figures 7A–D**). Previous studies have revealed that the Nr4a1-modulated expression of its target genes is primarily ascribed to its transcriptional activity (19, 22). Hence, to dissect the potential mechanism underlying the effect of Lcn10 deficiency in Nr4a1 signaling, we determined the translocation of Nr4a1 in macrophages from WT and KO mice using immunofluorescence staining. Under basal status, Nr4a1 was distributed in cytosol and nucleus, which is similar in WT and KO macrophages (**Figure 7E**). However, when treated with LPS+IFNγ, WT-BMDMs showed a substantial increase in the nuclear translocation of Nr4a1 when compared with controls **(** **Figure 7E**). Notably, such nuclear translocation was attenuated in KO-BMDMs as more Nr4a1 was retained in the cytosol (**Figure 7E**). Consistently, qRT-PCR analysis validated that such distribution change affected the expression of several Nr4a1-targeted genes in KO-BMDMs treated with LPS+IFNγ: with down-regulation of anti-inflammatory gene (*Gdf3*, *Tgfbi*) (**Figure 7F**), whereas up-regulating pro-inflammatory gene *Rab4a* (**Figure 7F**). Accordingly, CsnB treatment partially improved cardiac function in Lcn10-KO mice under T2D conditions, yet to a lesser extent when comparing to WT groups (**Figures 7G, H**). Taken together, these data indicate that disruption of the Nr4a1 signal may contribute to Lcn10 KO-mediated inflammatory responses in macrophages and subsequent cardiac injury.

## Discussion

In the present study, we have elucidated that Lcn10 plays a critical role in the regulation of macrophage polarization and diabetes-induced cardiac dysfunction. Major findings include: 1) metabolic stress inhibits Lcn10 expression in macrophages; 2) Lcn10 deficiency skews macrophages towards a pro-inflammatory phenotype, exacerbates insulin resistance, and impairs cardiac contractile function during diabetes conditions; 3) the Nr4a1 signaling pathway is disrupted in Lcn10-KO macrophages, leading to augmented inflammation; and 4) treatment with Nr4a1 agonist, CsnB, alleviates pro-inflammatory response in macrophages and partially improves cardiac function when comparing Lcn10-KO mice to WT controls.

The lipocalins are a family of proteins that share several common molecular-recognition properties and exhibit great functional diversity (43). For example, Lcn2, also referred to as neutrophil gelatinase-associated lipocalin (NGAL), has been well-investigated in metabolic disorders, and is characterized as a critical pro-inflammatory mediator during inflammation-associated diseases (40–42, 49–51). It is important to mention here, through sequencing peripheral blood RNA from 129 representative subjects, Tsalik *et al.* reported that Lcn2 was significantly higher in sepsis non-survivors than sepsis survivors and conversely, Lcn10 was increased dramatically in sepsis survivors than in non-survivor counterparts (52). However, Wang *et al.* recently showed that patients with sepsis-induced myocardial dysfunction (SIMD) displayed higher serum levels of Lcn10 than healthy donors, suggesting a compensatory mechanism in response to sepsis (53). Collectively, these previous observations implicate that Lcn10 may function as an anti-inflammatory mediator during the process of inflammation. Along this line, we provide a strong evidence in this study showing that Lcn10 is remarkably downregulated in macrophages in response to a variety of inflammatory stimuli (**Figures 1A–D**). Furthermore, Lcn10 deficiency potentiates pro-inflammatory gene expression in BMDMs upon stimulation with either LPS+IFNγ or palmitate (**Figures 2E–H**; **Supplementary Figures S1A–F, S2A–I**), whereas IL-4-induced anti-inflammatory gene expression is greatly suppressed (**Figures 2I–L**).

As the most abundant immune cells in the heart, macrophages account for 6–8% of non-cardiomyocytes with remarkable plasticity in phenotype and function (54). It has been well appreciated that macrophages play a critical role in cardiac remodeling in diabetes-induced heart dysfunction (5, 8). In the setting of diabetes, several pro-inflammatory factors, including hyperglycemia, hyperlipidemia, and hyperinsulinemia, are upregulated and promote increased secretion of cytokines, chemokines, and exosomes which consequently, contribute to the decline of cardiac resident macrophages and promote the accumulation of pro-inflammatory monocytes/macrophages in the heart, leading to the development of cardiomyopathy (16). Furthermore, these inflammatory cytokines such as IL-6, IL-1β, and TNFα released by macrophages can impair cardiomyocyte contractility, induce cardiac fibrosis and cardiac cell death, resulting in further adverse remodeling (10, 55, 56). In line with these previous findings, our results presented in this study clearly demonstrate a substantial increase of Ly6C^+^CD206^-^ pro-inflammatory macrophages in the hearts of Lcn10-KO mice under diabetic conditions, which largely contributes to the exacerbated cardiac dysfunction. In addition, Lcn10-KO macrophages exhibit higher expression of cytokines/chemokines (i.e., IL-6, IL-1β, and CCL2) than wild-type controls, which are also major culprits for the development of cardiomyopathy during diabetes. More importantly, adoptive transfer of Lcn10-KO bone marrow cells into X-ray irradiated mice (**Figure 5**) further validate such critical contributions of pro-inflammatory macrophages and their released cytokines/chemokines to the pathogenesis of diabetic cardiomyopathy.

With respect to how Lcn10 regulates macrophage phenotype, our RNA sequencing analysis reveals that Nr4a1 signaling cascades are dys-regulated in Lcn10-KO macrophages. We further observed that (LPS+IFNγ)-stimulated nuclear translocation of Nr4a1 was disrupted in Lcn10 deficiency macrophages, compared to WT-cells (**Figure 7E**). Currently, Nr4a1 is well characterized to have robust anti-inflammatory effects though directly targeting gene expression *via* its nuclear translocation and thereby, function as an either transcriptional activator or repressor (19, 22). Therefore, the mechanism underlying pro-inflammatory phenotype displayed in Lcn10-null macrophages could be associated with the disruption of Nr4a1-mediated anti-inflammatory signaling pathway. This explanation is further strongly supported by our data presented in **Figures 7A–D** showing that treatment of Lcn10-KO macrophages with Nr4a1 agonist remarkedly suppresses pro-inflammatory response.

As for how loss of Lcn10 impairs Nr4a1 translocation to nuclei in macrophages, it remains unclear in the present study. Considering that multiple molecular recognition properties shared by the lipocalin family proteins include ligand binding, macromolecular complexation, and the binding of cell surface receptors (57, 58), two possibilities could be speculated to explain how Lcn10 affects Nr4a1 trafficking in macrophages. First, Lcn10 may directly interact with Nr4a1 in the cytosol of macrophages where it functions as a chaperon protein and facilitates Nr4a1 translocation to the nuclei upon metabolic stress. Accordingly, Lcn10 deficiency disrupts such nuclear transfer of Nr4a1 and thus, augments inflammatory response to stress stimuli. Second, Lcn10 may have an autocrine effect and acts on the surface receptor of macrophages to activate its downstream signaling that promotes Nr4a1 nuclear translocation. In this regard, the absence of Lcn10 in macrophages would certainly limit Nr4a1 activation upon stress conditions. Future studies using a gain-of-function approach will be needed to shed light on these mechanisms.

## Conclusions

**Figure 8:**
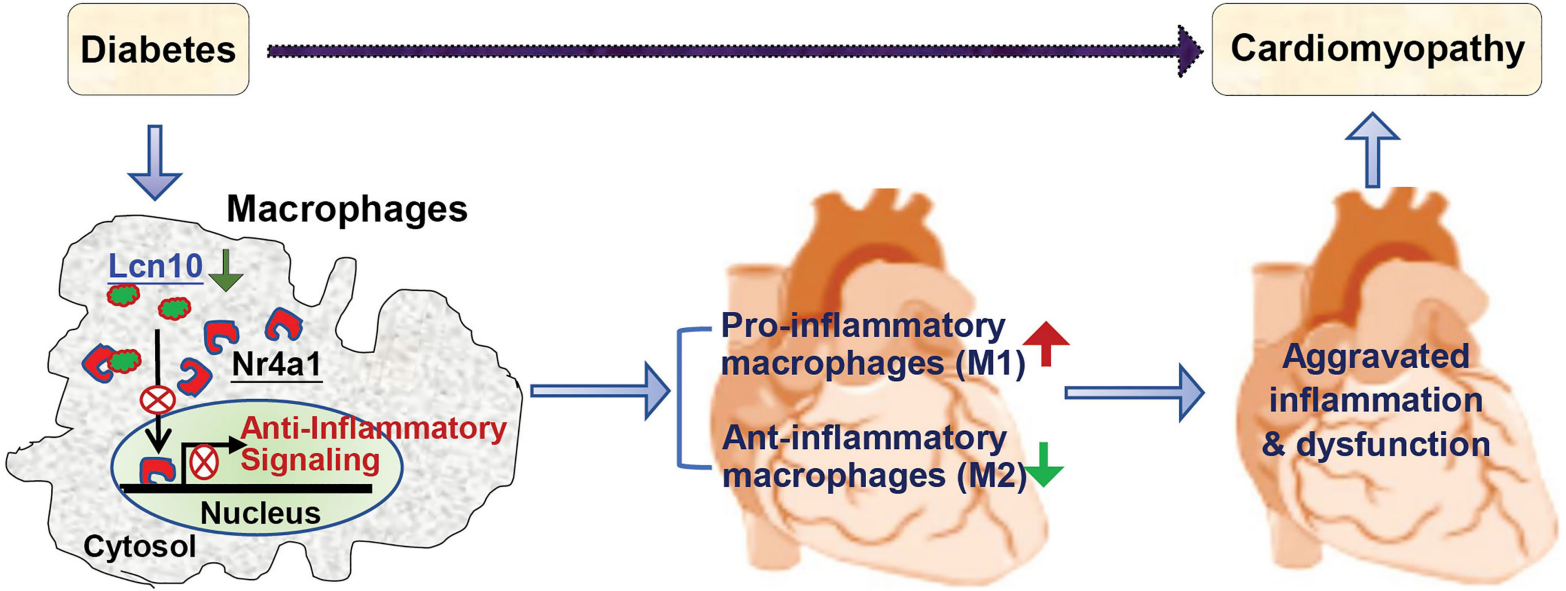
Scheme depicting that absence or reduction of Lcn10 expression in macrophages impairs Nr4a1 nuclear translocation, leading to increased pro-inflammatory macrophages and decreased anti-inflammatory macrophages accumulated in the heart and consequently, aggravated cardiac inflammation and dysfunction during diabetes.

This study, using a loss-of-function approach, elucidates a novel mechanism underlying the development of diabetic cardiomyopathy (**Figure 8**). This is associated with a reduction of Lcn10 expression in macrophages, resulting in: 1) an exacerbated inflammation through disrupting Nr4a1 signal, 2) a high ratio of pro-/anti-inflammatory macrophage population accumulated in the heart during diabetes. As a consequence, cardiac dysfunction is aggravated. Thus, any strategies that elevation of Lcn10 expression/activity in macrophages would possess therapeutic potential of diabetes-induced low-grade inflammation and concomitant cardiomyopathy.

## Data Availability Statement

The original contributions presented in the study are included in the article/**Supplementary Material**. Further inquiries can be directed to the corresponding author.

## Ethics Statement

The animal study was reviewed and approved by University of Cincinnati Animal Care and Use Committee.

## Author Contributions

QL designed and performed experiments, analyzed data, and wrote the manuscript. YL and ZL critically reviewed the manuscript. YL assisted with various experiments (particular flow cytometry) and help to analyze data (**Figures 4A–D** and **Supplementary Figure S3**). WH and XW performed echocardiography; WH and QL analyzed the echocardiography data. XW performed retro-orbital injection for bone marrow transplantation in the mice. JC analyzed RNA-sequencing data. YF, TP, SS, YW, and G-CF helped with experimental design, data analysis and critically reviewed the manuscript. G-CF analyzed results, reviewed/edited the manuscript, provided financial and administrative support, and gave final approval of the manuscript.

## Funding

This study was supported by National Institutes of Health (NIH) grants R01 (GM-126061, GM-132149, and HL-160811 to G-CF), and American Heart Association (AHA) Post-doctoral Fellowship Award (# 827586 to YL). SS has received support from National Institutes of Health grants (R01 HL130356, R01 HL105826, R01 AR078001, and R01 HL143490), American Heart Association, Institutional Undergraduate Student (19UFEL34380251), Transformation (19TPA34830084) awards, the PLN Foundation (PLN crazy idea) awards, as well as Novo Nordisk, AstraZeneca, MyoKardia, Merck and Amgen. The funders were not involved in the study design, collection, analysis, interpretation of data, the writing of this article or the decision to submit it for publication.

## Conflict of Interest

SS provided consulting and collaborative research studies to the Leducq Foundation (CURE-PLAN), Red Saree Inc., Greater Cincinnati Tamil Sangam, AavantiBio, Pfizer, Novo Nordisk, AstraZeneca, MyoKardia, Merck and Amgen.

The remaining authors declare that the research was conducted in the absence of any commercial or financial relationships that could be construed as a potential conflict of interest.

## Publisher’s Note

All claims expressed in this article are solely those of the authors and do not necessarily represent those of their affiliated organizations, or those of the publisher, the editors and the reviewers. Any product that may be evaluated in this article, or claim that may be made by its manufacturer, is not guaranteed or endorsed by the publisher.
