# Supplementary material for: Loss of Lipocalin 10 Exacerbates Diabetes-Induced Cardiomyopathy via Disruption of Nr4a1-Mediated Anti-Inflammatory Response in Macrophages
Source: Front Immunol. 2022 Jun 10;13:930397. doi: 10.3389/fimmu.2022.930397 (PMC9226549; doi:10.3389/fimmu.2022.930397)
Supplement: Supplementary file 1 [file DataSheet_1.docx]

Supplementary Material

## Supplementary Figure S1

**
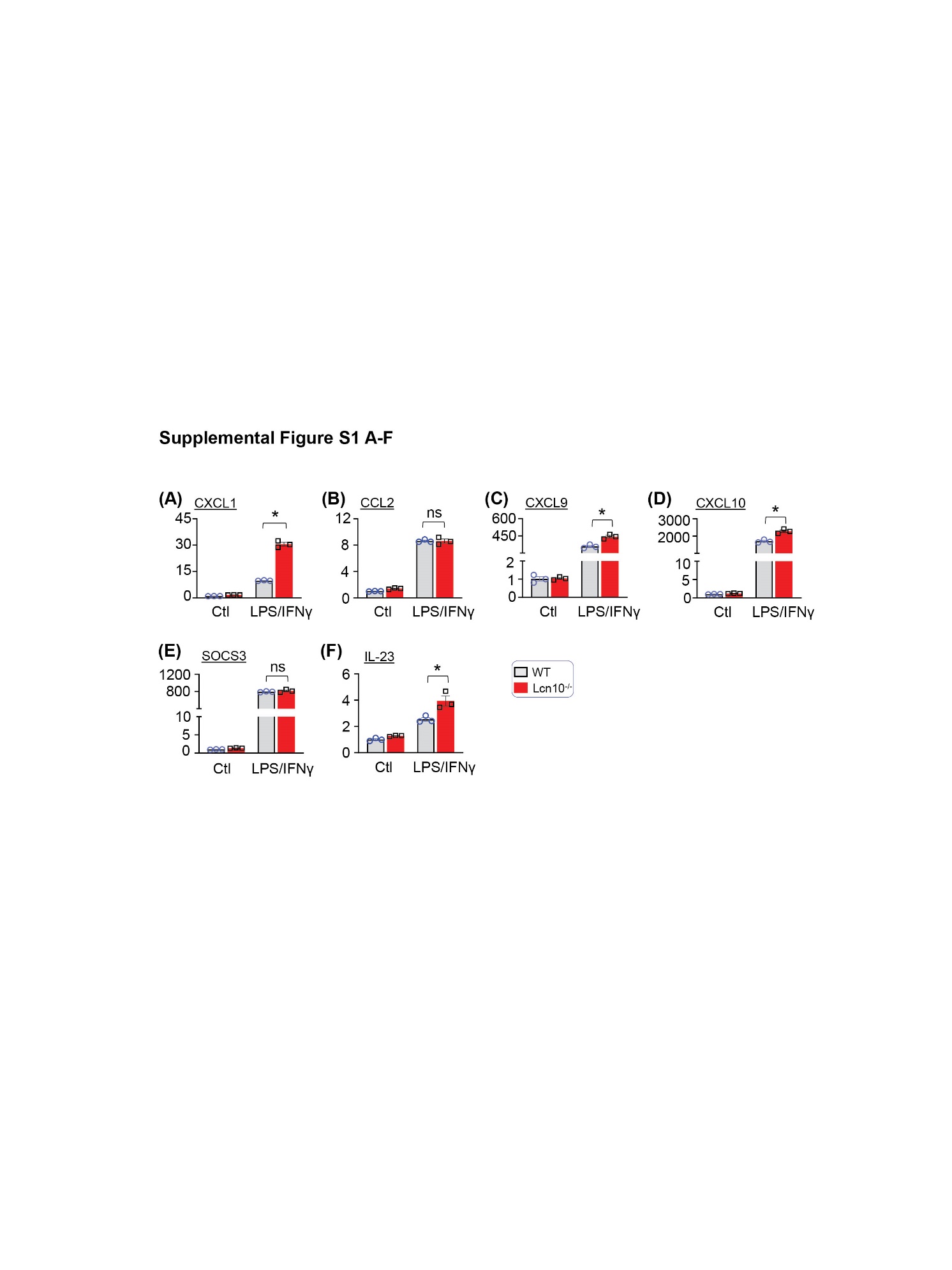
**

**Supplementary Figure S1.** **(A-F)** Gene expression levels of pro-inflammatory marker genes (CXCL1, CCL2, CXCL9, CXCL10, SOCS3, IL-23) as measured by qRT-PCR in BMDMs from WT and Lcn10-KO mice stimulated with LPS (10 ng/ml) + IFN-γ (10 ng/ml) for 6 hours (* *P* < 0.05, n=3 samples per group). All data are shown as mean ± SEM and analyzed by two-way ANOVA.

## Supplementary Figure S2


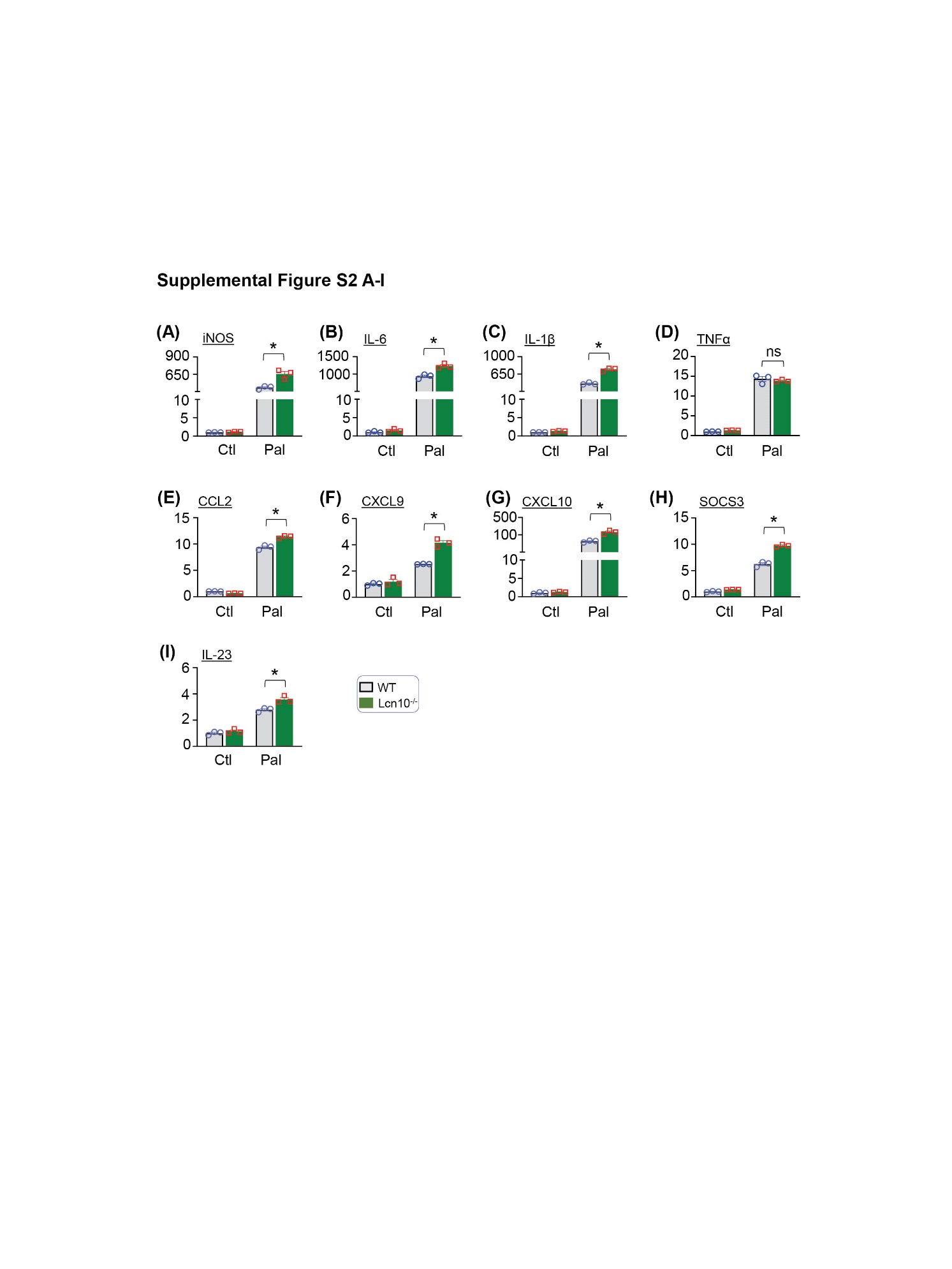


**Supplementary Figure S2.** **(A-I)** Gene expression levels of pro-inflammatory marker genes (iNOS, IL-6, IL-1β, TNFα, CCL2, CXCL9, CXCL10, SOCS3, IL-23), as measured by qRT-PCR in BMDMs from WT and Lcn10-KO mice, stimulated with palmitate (0.5 mM) for 6 hours (* *P* < 0.05, n=3 samples per group). All data are shown as mean ± SEM and analyzed by two-way ANOVA.

## Supplementary Figure S3

##
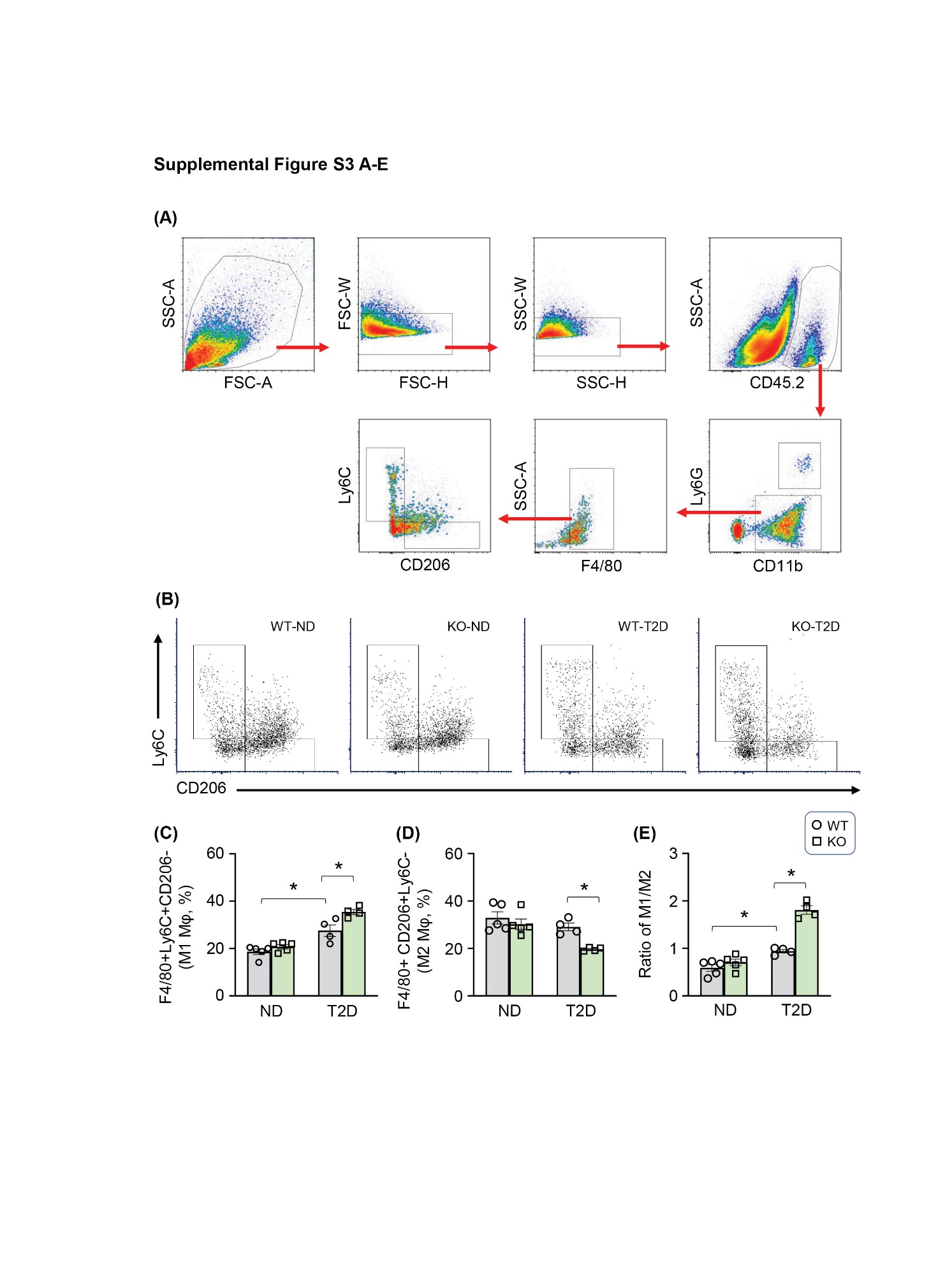


**Supplementary Figure S3.** **(A)** Flow cytometry gating strategy for analysis of cardiac leukocytes. **(B-E)** Representative flow cytometry plots **(B)** and quantification of cardiac pro-inflammatory M1-like macrophages (F4/80+Ly6C+CD206-) **(C)**, anti-inflammatory M2-like macrophages (F4/80+Ly6C-CD206+) **(D)** and the ratio of M1/M2 **(E)** (* *P* < 0.05, n=4-5 mice per group). All data are shown as mean ± SEM and analyzed by two-way ANOVA.

## Supplementary Figure S4


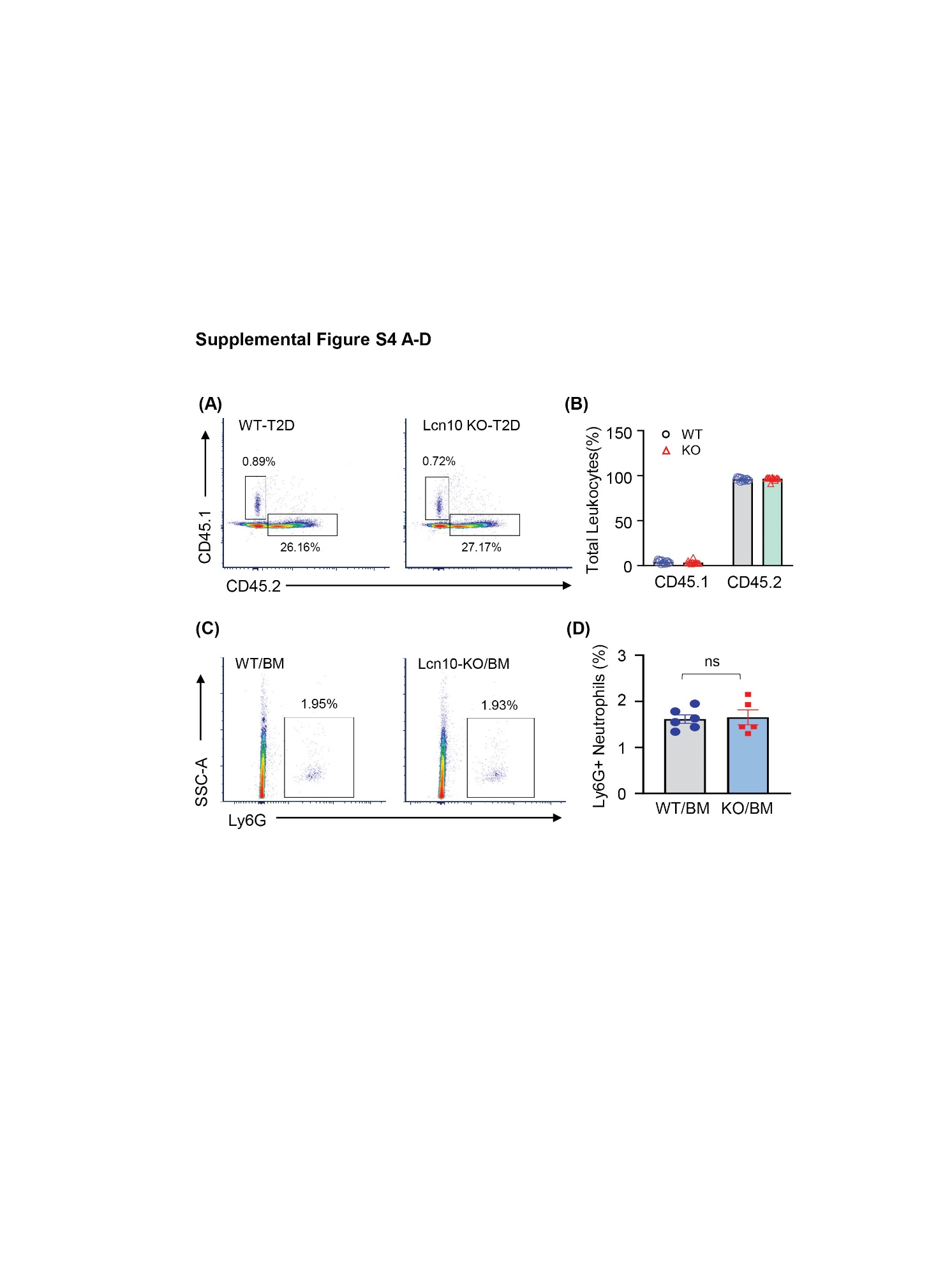


**Supplementary Figure S4.** **(A and B)** Transplantation of CD45.2 bone marrow cells from WT and Lcn10-KO mice into CD45.1 recipient mice. CD45.1 recipient cells were almost entirely abolished after whole-body irradiation, and CD45.2 donor cells were successfully reconstituted in the hearts (n=13 mice per group). Note the quantification data was calculated as [CD45.1/(CD45.1+CD45.2)] *100% and [CD45.2/(CD45.1+CD45.2)] *100%. **(C, D)** Ly6G neutrophils showed no difference in the T2D heart injected with WT and Lcn10-KO bone marrow cells (* *P* < 0.05, n=5-6 mice per group). All data are presented as mean ± SEM and analyzed by student’s t-test.

## Supplementary Table S1

| **qRT-PCR primers** | | |
| --- | --- | --- |
| Genes | Primer sequences (5′ → 3′) | Amplicon size (bp) |
| GAPDH | Forward: TGCACCACCAACTGCTTAGC  Reverse: GGCATGGACTGTGGTCATGAG | 87 |
| Lcn10 | Forward: CATCTTCCGATCCCAGAAAA  Reverse: CTCAGCCTCCAGTGAAGTCC | 97 |
| Lcn2 | Forward: GCCACCACGGACTACAACC  Reverse: GCTCCTTGGTTCTTCCATACAGG | 100 |
| NR4A1 | Forward: TTGAGTTCGGCAAGCCTACC  Reverse: CCGTCCATGAAGGTGCTGAAG | 94 |
| iNOS | Forward: CAGCTGGGCTGTACAAACCTT  Reverse: CATTGGAAGTGAAGCGTTTCG | 95 |
| IL6 | Forward: CTGCAAGAGACTTCCATCCAG  Reverse: AGTGGTATAGACAGGTCTGTTGG | 131 |
| IL1B | Forward: GCAACTGTTCCTGAACTCAACT  Reverse: ATCTTTTGGGGTCCGTCAACT | 89 |
| CCL2 | Forward: TTAAAAACCTGGATCGGAACCAA  Reverse: GCATTAGCTTCAGATTTACGGGT | 121 |
| CXCL9 | Forward: GGAGTTCGAGGAACCCTA GTG  Reverse: GGGATTTGTAGTGGATCGTGC | 82 |
| CXCL10 | Forward: CCAAGTGCTGCCGTCATTTTC  Reverse: GGCTCGCAGGGATGATTTCAA | 157 |
| TNF | Forward: CAGGCGGTGCCTATGTCTC  Reverse: CGATCACCCCGAAGTTCAGTAG | 89 |
| SOCS3 | Forward: ATGGTCACCCACAGCAAGTTT  Reverse: TCCAGTAGAATCCGCTCTCCT | 145 |
| IL-23 | Forward: GCTGTGCCTAGGAGTAGCAG  Reverse: TGGCGTTTGTCCTTGAGTCC | 203 |
| CXCL1 | Forward: CTGGGATTCACCTCAAGAACATC  Reverse: CAGGGTCAAGGCAAGCCTC | 117 |
| Arg1 | Forward: TCATGGAAGTGAACCCAACTCTTG  Reverse: TCAGTCCCTGGCTTATGGTTACC | 127 |
| Chil3 | Forward: AGAAGGGAGTTTCAAACCTGGT  Reverse: GTCTTGCTCATGTGTGTAAGTGA | 109 |
| Clec10a | Forward: TGAGAAAGGCTTTAAGAACTGGG  Reverse: GACCACCTGTAGTGATGTGGG | 101 |
| Retnla | Forward: TGCCAATCCAGCTAACTATCCC  Reverse: CAGTGGTCCAGTCAACGAGT | 197 |
| Gdf3 | Forward: CCACCCCACCTTTGGCTTAT  Reverse: GTAAGCTCACCAAGGGGTCC | 113 |
| Id3 | Forward: CTTACCCTGAACTCAACGCCT  Reverse: CAGCGTGTGCTAGCTCTTCA | 87 |
| Itga6 | Forward: TGCAGAGGGCGAACAGAAC  Reverse: GCACACGTCACCACTTTGC | 175 |
| Mid1 | Forward: CTGTGACGGCACCTGTCTC  Reverse: AAACGGCTGACTGTTGGTCTT | 205 |
| Notch4 | Forward: CTCTTGCCACTCAATTTCCCT  Reverse: TTGCAGAGTTGGGTATCCCTG | 200 |
| Smad6 | Forward: GCAACCCCTACCACTTCAGC  Reverse: GTGGCTTGTACTGGTCAGGAG | 90 |
| Tgfbi | Forward: CAGCACGGCCCCAATGTAT  Reverse: GGGACCTTTTCATATCCAGGACA | 149 |
| Rab4a | Forward: TTCTTGGTCATCGGAAATGCG  Reverse: TCTTTGAGCCAAATTCCATTCCT | 115 |
| Ednrb | Forward: GTGGCTTCTTGGGGGTATGG  Reverse: TCTTAGTGGGTGGCGTCATTA | 102 |
| Hpse | Forward: ACCGACGACGTGGTAGACTT  Reverse: GCAGGAGATAAGCCTCTAGCC | 167 |
| Clec2d | Forward: GGTTTGACAACCAGGATGAGC  Reverse: TCTCCCCGGATGGGAATCG | 148 |
| **Genotyping primers** | | |
| Wildtype (WT) | Forward: GACACCTCCACAAAGGAGCTGAGG  Reverse: CCCTGTCCCTGACTAGAATGAAGAGG | 313 |
| Lcn10 Knockout (KO) | Forward: GACACCTCCACAAAGGAGCTGAGG  Reverse: CCCTGTCCCTGACTAGAATGAAGAGG | 727 |

## Supplementary Table S2

| Reagent Name | Manufacturer | Clone | Catalog # | Dilution |
| --- | --- | --- | --- | --- |
| CD45.2 Alexa Fluor 488 | BioLegend | 104 | 109816 | 1:50 |
| CD45.2 PerCp-Cy5.5 | BioLegend | 104 | 109828 | 1:50 |
| CD11b BV650 | BioLegend | M1/70 | 101259 | 1:50 |
| CD11b PE | BioLegend | M1/70 | 101208 | 1:50 |
| Ly6G APC | BioLegend | 1A8 | 127614 | 1:50 |
| F4/80 PE | BioLegend | BM8 | 123110 | 1:100 |
| F4/80 APC | BioLegend | BM8 | 123116 | 1:50 |
| F4/80 Pacific Blue | BioLegend | BM8 | 123124 | 1:50 |
| CCR2 PE-Cy7 | BioLegend | SA203G11 | 150612 | 1:50 |
| Ly6C PE-Dazzle 594 | BioLegend | HK1.4 | 128044 | 1:500 |
| CD206 BV605 | BioLegend | C068C2 | 141721 | 1:50 |
| CD206 BV421 | BioLegend | C068C2 | 141717 | 1:100 |
| CD38 PE-Cy7 | Invitrogen | 90 | 25-0381-82 | 1:100 |
| CD45.1 BV785 | BioLegend | A20 | 110743 | 1:50 |

## Supplementary Table S3

| Parameter | Units | WT-ND | KO-ND | WT-T2D | KO-T2D |
| --- | --- | --- | --- | --- | --- |
| Diameter;s | mm | 2.72 ± 0.13 | 2.83 ± 0.04 | 2.66 ± 0.07 | 3.25 ± 0.12 # |
| Diameter;d | mm | 3.96 ± 0.15 | 4.07 ± 0.06 | 3.52 ± 0.08 | 4.03 ± 0.12 # |
| Volume;s | µL | 28.42 ± 3.41 | 30.31 ± 1.02 | 26.39 ± 1.57 | 43.35 ± 4.03 # |
| Volume;d | µL | 69.72 ± 6.51 | 73.21 ± 2.38 | 52.03 ± 2.69 | 72.10 ± 4.97 # |
| Stroke Volume | µL | 41.29 ± 3.30 | 42.90 ± 1.69 | 25.63 ± 1.18 * | 28.75 ± 1.35 |
| Ejection Fraction | % | 59.85 ± 1.47 | 58.56 ± 0.94 | 49.39 ± 0.63 * | 40.43 ± 1.66 # |
| Fractional Shortening | % | 31.46 ± 0.96 | 30.61 ± 0.65 | 24.40 ± 0.34 * | 19.48 ± 0.89 # |
| Cardiac Output | mL/min | 22.95 ± 1.57 | 22.65 ± 1.08 | 12.56 ± 0.60 * | 11.72 ± 0.68 |

* *P* < 0.05, when comparing WT ND to WT T2D

^#^ *P* < 0.05, when comparing WT T2D to KO T2D

## Supplementary Table S4

| Parameter | Units | WT-T2D | KO-T2D |
| --- | --- | --- | --- |
| Diameter;s | mm | 2.36 ± 0.06 | 2.52 ± 0.08 |
| Diameter;d | mm | 3.53 ± 0.08 | 3.54 ± 0.08 |
| Volume;s | µL | 19.49 ± 1.13 | 22.93 ± 1.81 |
| Volume;d | µL | 52.15 ± 2.78 | 52.36 ± 2.83 |
| Stroke Volume | µL | 32.66 ± 2.15 | 29.42 ± 1.25 |
| Ejection Fraction | % | 62.51 ± 1.66 | 56.39 ± 1.40 * |
| Fractional Shortening | % | 33.05 ± 1.19 | 28.82 ± 0.90 * |
| Cardiac Output | mL/min | 15.32 ± 1.53 | 13.53 ± 0.71 |

* *P* < 0.05, when comparing WT T2D to KO T2D

## Supplementary Table S5

| Parameter | Units | WT-T2D Vehicle | KO-T2D Vehicle | WT-T2D CsnB | KO-T2D CsnB |
| --- | --- | --- | --- | --- | --- |
| Diameter;s | mm | 2.75 ± 0.08 | 3.28 ± 0.17 | 2.76 ± 0.17 | 3.38 ± 0.16 |
| Diameter;d | mm | 3.64 ± 0.09 | 4.00 ± 0.16 | 3.92 ± 0.19 | 4.35 ± 0.15 |
| Volume;s | µL | 28.49 ± 2.15 | 44.45 ± 5.27 | 29.14 ± 4.34 | 47.28 ± 5.26 |
| Volume;d | µL | 56.12 ± 3.17 | 70.72 ± 6.44 | 67.58 ± 7.77 | 85.94 ± 7.01 |
| Stroke Volume | µL | 27.63 ± 1.02 | 26.27 ± 1.54 | 38.44 ± 3.53 | 38.67 ± 2.04 ^#^ |
| Ejection Fraction | % | 49.44 ± 0.96 | 37.72 ± 2.11 | 57.34 ± 1.67 * | 45.42 ± 2.12 ^#^ |
| Fractional Shortening | % | 24.49 ± 0.53 | 17.96 ± 1.10 | 29.67 ± 1.02 * | 22.46 ± 1.22 ^#^ |
| Cardiac Output | mL/min | 13.48 ± 0.51 | 10.76 ± 0.43 | 16.29 ± 1.43 * | 14.81 ± 1.09 ^#^ |

* *P* < 0.05, when comparing WT T2D vehicle to WT-T2D CsnB

^#^ *P* < 0.05, when comparing KO T2D vehicle to KO-T2D CsnB
